# Does vancomycin resistance increase mortality in *Enterococcus faecium* bacteraemia after orthotopic liver transplantation? A retrospective study

**DOI:** 10.1186/s13756-020-0683-3

**Published:** 2020-01-31

**Authors:** S. Dubler, M. Lenz, S. Zimmermann, D. C. Richter, K. H. Weiss, A. Mehrabi, M. Mieth, T. Bruckner, M. A. Weigand, T. Brenner, A. Heininger

**Affiliations:** 1 Department of Anaesthesiology, Heidelberg University Hospital, Im Neuenheimer Feld 110, D-69120 Heidelberg, Germany; 2 Department of Anaesthesiology, Intensive Care and Emergency Medicine, Asklepios Clinics Hamburg, AK Wandsbek, Hamburg, Germany; 3 Department of Infectious Diseases, Medical Microbiology and Hygiene, Division Bacteriology, Heidelberg University Hospital, Heidelberg, Germany; 4 Department of Internal Medicine, Heidelberg University Hospital, Heidelberg, Germany; 5 Department of Visceral and Transplant Surgery, Heidelberg University Hospital, Heidelberg, Germany; 6 Institute for Medical Biometry and Informatics, University of Heidelberg, Heidelberg, Germany; 7 Division Hospital and Environmental Hygiene Department of Infectious Diseases, Medical Microbiology and Hygiene, Heidelberg University Hospital, Heidelberg, Germany

**Keywords:** Enterococci, Bacteraemia, Mortality, Vancomycin resistance, Liver transplantation

## Abstract

**Background:**

The relevance of vancomycin resistance in enterococcal blood stream infections (BSI) is still controversial. Aim of this study was to outline the effect of vancomycin resistance of *Enterococcus faecium* on the outcome of patients with BSI after orthotopic liver transplantation (OLT).

**Methods:**

The outcome of OLT recipients developing BSI with vancomycin-resistant (VRE) versus vancomycin-susceptible *Enterococcus faecium* (VSE) was compared based on data extraction from medical records. Multivariate regression analyses identified risk factors for mortality and unfavourable outcomes (defined as death or prolonged intensive care stay) after 30 and 90 days.

**Results:**

Mortality was similar between VRE- (*n* = 39) and VSE- (*n* = 138) group after 30 (*p* = 0.44) or 90 days (*p* = 0.39). Comparable results occurred regarding unfavourable outcomes. Mean SOFA_Non-GCS_ score during the 7-day-period before BSI onset was the independent predictor for mortality at both timepoints (HR 1.32; CI 1.14–1.53; and HR 1.18; CI 1.08–1.28). Timely appropriate antibiotic therapy, recent ICU stay and vancomycin resistance did not affect outcome after adjusting for confounders.

**Conclusion:**

Vancomycin resistance did not influence outcome among patients with *Enterococcus faecium* bacteraemia after OLT. Only underlying severity of disease predicted poor outcome among this homogenous patient population.

**Trial registration:**

This study was registered at the German clinical trials register (DRKS-ID: DRKS00013285).

## Background

For decades, enterococci were considered to be harmless inhabitants of the human gastrointestinal tract, only rarely causing opportunistic infections in critically ill patients. Currently, they have been studied to be among leading pathogens of nosocomial infections, and thus they are a major international health burden [1–3]. Enterococci are one of the most common isolated species causing nosocomial blood stream infections (BSI) in intensive care units, ranking second in the United States [4] and third in European intensive care units [5]. Crude mortality rates are between 20 and 50% in these patient populations [2, 6, 7].

Treatment of enterococcal blood stream infections has become more difficult due to the increase of multidrug resistance. Prevalence of vancomycin-resistant enterococci (VRE) has increased worldwide. In a US study, up to 80% of *Enterococcus faecium* isolates proved to be vancomycin-resistant [8]. The proportion of vancomycin resistance in enterococcal BSI rose from 5.9% in 2007 to 16.7% in 2016 [9] in German ICUs. The independent impact of this increasing vancomycin resistance on patients’ outcome is still a matter of debate. Studies show conflicting results in terms of mortality of patients with VRE bacteraemia compared to cases caused by vancomycin-susceptible enterococci (VSE). Previous studies and systematic reviews were conducted prior to the availability of effective antibiotics against VRE [10–12]. Moreover, studies often included heterogeneous patient populations and did not adjust for disease severity [13]. Finally, most studies that compared outcomes between patients suffering from infections with multidrug-resistant pathogens and those with susceptible microorganisms failed to consider important confounders [13, 14].

Patients after solid organ transplantation are at increased risk for nosocomial infections due to multidrug-resistant bacteria [15] and particularly to VRE bacteraemia [10, 16, 17]. Mortality rates up to 46% in the context of VRE bacteraemia have been described in these patients [10]. However, investigations to outline the independent effect of VRE on the outcome of patients after orthotopic liver transplantation (OLT) are lacking. Thus, we performed a retrospective data analysis in our liver transplantation centre of all patients, who developed BSI after OLT due to vancomycin-resistant versus vancomycin-susceptible *E. faecium* isolates within an 11-year observation period.

## Methods

### Study design and population

**Fig. 1:**
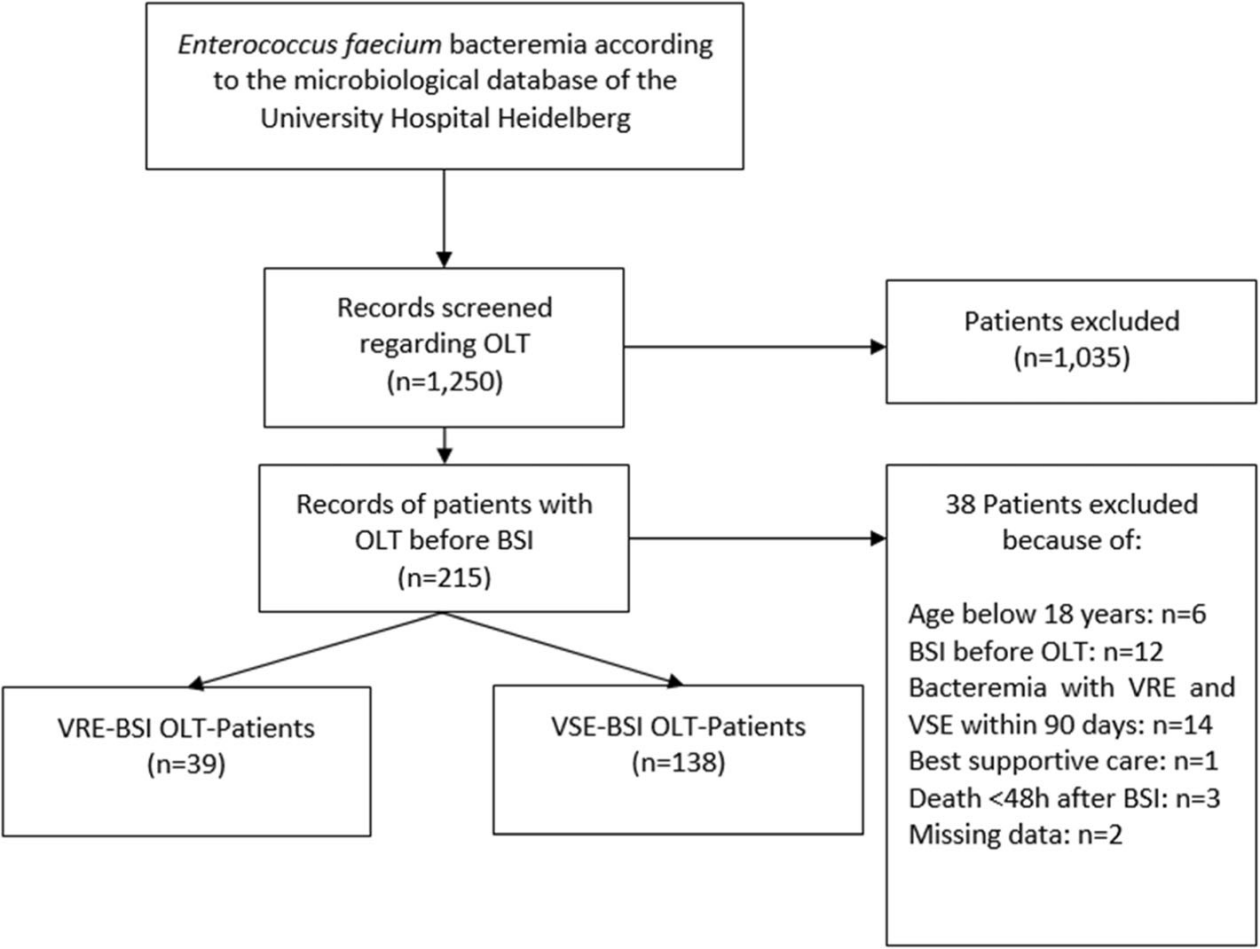
Flow diagram for this study. Abbreviations: OLT, orthotopic liver transplantation; BSI, blood stream infection; VRE, vancomycin-resistant *Enterococcus faecium*; VSE, vancomycin-susceptible *E. faecium*

This study was conducted as a monocentric, retrospective study at the University Hospital Heidelberg (Germany). It was approved by the local ethics committee of the Medical Faculty of the University of Heidelberg (S-407/2017) and was registered at the German clinical trials register (DRKS-ID: DRKS00013285). Adult liver transplant recipients having one or more positive blood culture with *E. faecium* from January 1st, 2006 to December 31st, 2016 were eligible. The treatment of patients was based on the Heidelberger manual of OLT. This includes a standardised immunosuppression regimen consisting of corticosteroids, calcineurin inhibitors (tacrolimus or ciclosporin) and mycophenolat-mofetil (MMF). The exclusion criteria were: patients aged < 18 years, BSI occurring prior to OLT, BSI with both, vancomycin-resistant and vancomycin-susceptible *E. faecium* within the same 90- days period, patients with best supportive care and death < 48 h after BSI and patients with missing relevant data. Fig. 1 summarizes inclusion and exclusion criteria.

**Fig. 2:**
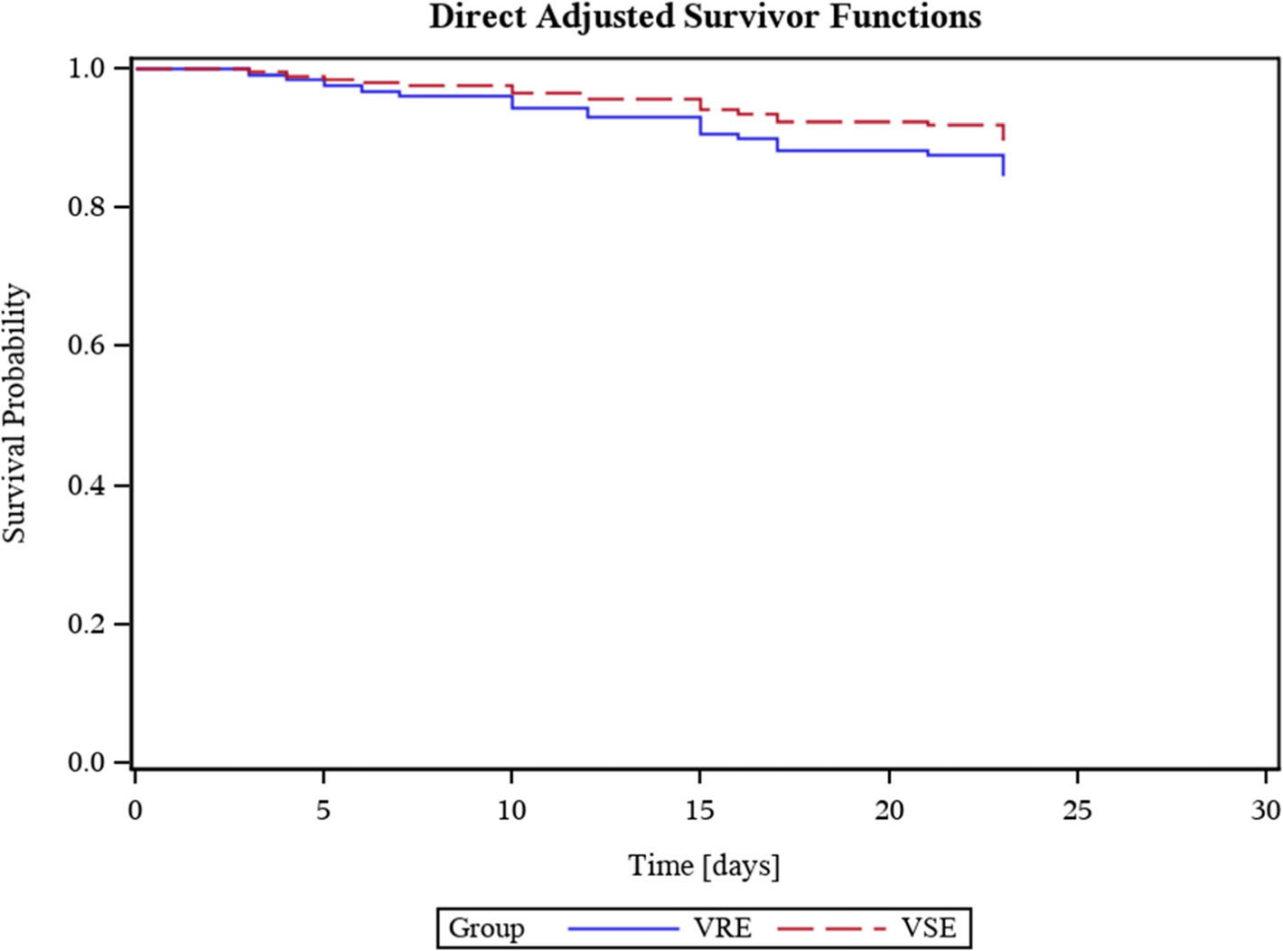
Kaplan-Meier survival curves after bloodstream infections with vancomycin-resistant *E. faecium* (VRE) versus vancomycin-susceptible *E. faecium* (VSE) following orthotopic liver transplantation (OLT). Data were adjusted for confounders

### Patient data

All patients of the University Hospital Heidelberg, Germany, with VRE or VSE bacteraemia during the study period were identified using the Swisslab microbiological laboratory database (Swisslab DITS GmbH, Berlin, Germany). Subsequently, a trained researcher screened the electronic medical records (ISH®, SAP, Walldorf, Germany) of all VRE and VSE-bacteraemia patients, whether they had had a liver transplantation before enterococcal BSI emerged.

The following data were extracted: demographics, Model for End-stage Liver Disease (MELD) score prior to the most recent liver transplantation before BSI, length of hospital stay pre- and post-BSI onset (ICU, ward, rehabilitation centre, at home), antibiotic therapy, surgical or other interventions, number of re-transplantations and control of the infectious focus (Table 1). To assess the acute status of illness, the sequential organ failure assessment (SOFA) score [18] was calculated. Since assessment and documentation of the Glasgow Coma Score (GCS) in ICU patients is frequently uncertain and/or incomplete, we defined a modified SOFA_Non-GCS_ score for our data analyses, excluding the item GCS [19, 20]. SOFA_Non-GCS_ scores were assessed during the 7-day period before BSI onset.

**Table 1:** Baseline characteristics of subjects included in this study

	VRE-BSI (*n* = 39)	VSE-BSI (*n* = 138)	*p*
Age [Years]	53.7 ± 10.9	52.2 ± 10.7	0.408
Females	11 (28.2)	40 (29.0)	0.924
Re-OLT before BSI onset?			0.391
No	18 (46.2)	60 (43.5)	
One	21 (53.9)	68 (49.3)	
Two	0 (0)	7 (5.1)	
Three	0 (0)	3 (2.2)	
MELD Score last OLT*	27.4 ± 10.2	22.9 ± 10.8	0.019
Interventions (d-30 until BSI onset) to:			
increase arterial perfusion of the liver	0.6 ± 0.9	0.3 ± 0.7	0.023
abscess drainage	0.2 ± 0.5	0.2 ± 0.6	0.927
biliary drainage	0.5 ± 1.1	0.8 ± 1.2	0.251
Laparotomies	0.4 ± 0.7	0.5 ± 1.0	0.741
Total interventions (d-30 until BSI onset)	2.3 ± 1.8	2.5 ± 1.8	0.612
Days from last LTX until BSI**	25 [1–9883]	21 [1–4423]	0.719
Mean SOFANon-_GCS_ (d-7 until d-1 before BSI) ***	8.1 ± 4.6	7.4 ± 5.1	0.373
Days in ICU before BSI onset (within 7 days before BSI)	6.1 ± 3.0	5.1 ± 3.5	0.050
Days on ward before BSI onset (within 7 days before BSI)	1.6 ± 2.6	2.2 ± 3.1	0.204
Days at home before BSI onset (within 7 days before BSI)	0.3 ± 1.4	0.7 ± 1.8	0.057

Data are presented as n (%), mean ± standard deviation.

* *n* = 176 (1 missing)

**Values displayed as median [range]

*** *n* = 172 (5 missing)

Abbreviations: *VRE* vancomycin-resistant *Enterococcus faecium*, *VSE* vancomycin-susceptible *E. faecium*, *OLT* orthotopic liver transplantation, *ICU* intensive care unit, *BSI* bloodstream infection

To control for potentially confounding factors, four variables beyond vancomycin resistance that may mirror the acute status of illness before BSI onset and adequacy of treatment were included in a logistic regression model for multivariate analysis of mortality as well as for the combined endpoint of an unfavourable outcome as defined below. For this aim, we applied the following items: mean SOFA_Non-GCS_ score and number of days spent in the ICU within 7 days prior to enterococcal bacteraemia (from day − 7 to day − 1), definitive elimination of the BSI-causing infectious focus and adequacy of antibiotic treatment within 48 h post-BSI onset.

### Outcomes

Primary endpoint was mortality (from any cause) on days 30 and 90 post-BSI onset. As secondary endpoint, we classified the outcome of patients as favourable, i.e., stay at home, on the ward or in a rehabilitation centre, versus unfavourable, i.e., death or persistent stay in the ICU, at days 30 and 90 post-BSI onset.

### Microbiology

Microbial growth of blood culture bottles (BACTEC FX® Aerobic/F, Anaerobic/F, BD Diagnostics, Heidelberg, Germany) was detected by the BACTEC FX® automated blood culture system (BD Diagnostics, Heidelberg, Germany) and subsequently confirmed by Gram staining. Positive cultures were worked up according to approved in-hospital standard techniques. Real-time PCR for vanA and vanB genes was performed for all positive blood culture bottles with Gram-positive cocci in the smear stain as described previously. Therefore, glycopeptide resistance was usually detected on the day the blood culture signalled positive [21].

### Definitions

The term *enterococcal BSI* was restricted to the isolation of *E. faecium* in at least one blood culture taken for clinical diagnostics. *Days to appropriate antibiotic therapy* was defined as period from the sampling time of the first blood culture indicating *E. faecium* to the start of at least one intravenously applied antimicrobial substance with proven efficacy (according to susceptibility testing of the blood culture isolate). *Empiric antibiotic therapy* was defined as the start of an antibiotic therapy before definitive results of susceptibility testing were available. *Targeted antibiotic therapy* was treatment given after receipt of antimicrobial susceptibility testing results. *Definitive infectious focus elimination* was defined as procedures resulting in a complete removal of persisting infectious clusters (e.g. cholecystectomy in the case of acute cholecystitis), differentiated from procedures for focus control (e.g. drainages for the treatment of insufficiencies of the biliodigestive anastomosis) [22, 23]. Two examiners independently reviewed the complete medical records of the patients and classified whether or not definitive infectious focus elimination was achieved. *Polymicrobial bacteraemia* was defined as isolation of more than one pathogen in the same blood culture.

*Interventions on the biliary system* were defined as percutaneous transhepatic cholangiodrainage (PTCD), endoscopic retrograde cholangiopancreatography (ERCP) and other interventions concerning the biliodigestive anastomosis (BDA) or biliary system, e.g., stenting of the bile duct. Interventions to increase liver perfusion included dilatation of liver arteria(s), stenting of liver arteria(s), catheter for lysis or infusion therapy of vasodilating drugs, coiling of aneurysm(s) and coiling of arteria lienalis.

### Data analysis

The data were stored in an EXCEL file (Microsoft®, Redmond, WA, USA) and then imported into SAS 9.4Win for analysis (SAS Institute Inc., Cary, NC, USA). Findings were reported using the mean and standard deviation for continuous data and absolute and relative frequencies for categorical variables. Potential differences between the groups were calculated using the Mann-Whitney U-test or chi-square test, as appropriate. Kaplan Meier curves and corresponding log-rank tests were used to compare the course of survival. Cox-regression and binary logistic regression models were used to identify possible prognostic factors for mortality [Hazard Ratio (HR)] and unfavourable outcomes [Odds Ratio (OR)], respectively. For all analyses, a two-sided *p* value less than 0.05 was considered statistically significant. The reported *p* values are descriptive due to the explorative nature of the study.

## Results

During the 11-year observation period, we identified a total of 1250 patients with *E. faecium* BSI in our study centre; 177 of them were adult liver transplant recipients and were included in the final analysis (Fig. 1). Liver transplantations in these patients were performed between 1989 and 2016. In 39 of the 177 cases (21.5%), the *E. faecium* isolates were VRE, whereas 138/177 (78.5%) were VSE cases. The baseline characteristics of the patients included are presented in Table 1. In both groups, the majority of patients were male. Age and MELD scores at OLT were comparable between the VRE and VSE group.

There was no difference between groups regarding median time from last OLT to first enterococcal BSI or meanSOFANon-GCS scores from day − 7 to day − 1 before BSI. During the week prior to BSI onset, VRE patients tended to spend more time on the ICU than VSE patients [6.1 ± 3.0 days versus 5.1 ± 3.5 days for VRE and VSE patients in mean, respectively, (*p* = 0.05)]. However, the average number (mean) of interventions per patient to increase the arterial perfusion of the graft within 30 days before BSI was higher in VRE than in VSE patients. The proportion of BSI caused by more than one pathogen was low (17 of 177 cases) and similar in both groups (*p* = 0.29). See Additional file 1: Table S1 for a detailed list of co-pathogens. The by far most often observed focus of BSI was an intraabdominal infection; whereas other infection sites (such as urinary tract infections, primary blood stream infections, infections of unknown origin, others) occurred very rarely in the presented cohort of patients undergoing OLT (Additional file 1 Table S6 and Additional file 1: Table S7).

In the VRE and the VSE group, 6 of 39 (16.2%) and 22 of 138 (17.3%) patients, respectively, were treated empirically with effective antibiotics before receipt of the microbiological result (*p* = 0.88). Five of these six VRE-patients empirically treated with linezolid were known to be carrier of VRE. For targeted antibiotic therapy in the VRE group, linezolid was most frequently prescribed [31/39 patients (79.5%)], followed by tigecycline given in 6 of 39 (15.4%) patients. Vancomycin was the most applied antibiotic in the VSE group [88/138 (63.8%)], followed by linezolid [31/138 (22.5%)]. Details are given in Additional file 1**:** Table S2. Adequate antibiotic treatment according to the susceptibility testing of the detected *E. faecium* isolate within 48 h post-BSI onset was established in 21 of 39 (53.9%) patients in the VRE group and 83 of 138 (60.1%) patients in the VSE group (*p* = 0.48). All patients who did receive antibiotic therapy during the study period were treated with an appropriate agent according to resistance testing. See Additional file 1: Table S2 for details on antibiotic therapy. Infectious source control procedures were performed in all patients; however, definitive elimination of the infectious focus was achieved within 30 days after BSI onset in only 9 of 39 (23.1%) patients in the VRE group and 37 of 138 (26.8%) patients in the VSE group (*p* = 0.64). The number of performed interventions from BSI onset until day 30 did not differ significantly between the two study groups (*p* = 0.20); more details are presented in Additional file 1 Table S3.

Table 2 presents the unadjusted outcomes after VRE- versus VSE-BSI in our OLT recipients. The primary outcome did not differ between both groups with a crude mortality rate 30 days after first enterococcal bacteraemia of 15.4% (6/39) in the VRE group and 10.9% (15/138) in the VSE group (*p* = 0.44). See Fig. 2 for Kaplan-Meier survival curves. Similarly, after 90 days, 15 of 39 (38.5%) VRE patients and 43 of 138 (31.2%) VSE patients had died (*p* = 0.39).

**Table 2:** Unadjusted outcome analysis of patients with vancomycin-resistant *Enterococcus faecium* (VRE) versus vancomycin-susceptible *E. faecium* (VSE) bacteraemia

Variables	n	All patients *n* = 177	VRE *n* = 39	VSE *n* = 138	*p*
30-day Endpoints					
Favourable Outcome	175*	95/175 (54.3)	19/39 (48.7)	76/136 (55.9)	0.429
Crude mortality	177	21/177 (11.9)	6/39 (15.4)	15/138 (10.9)	0.441
90-day Endpoints					
Favourable Outcome	172**	91/172 (52.9)	20/39 (51.3)	71/133 (53.4)	0.817
Crude Mortality	177	58/177 (32.8)	15/39 (38.5)	43/138 (31.2)	0.391
Length of hospital stay until day 30 [days]	177		25.9 ± 8.5	24.9 ± 7.7	0.160
Stay in ICU until day 30 [days]	177		15.3 ± 12.3	13.5 ± 12.0	0.435
Ward stay until day 30 [days]	156***		12.6 ± 12.1	12.8 ± 10.7	0.958
Stay at home or in a rehabilitation unit until day 30 [days]	156***		1.1 ± 4.3	3.4 ± 6.3	0.022

Data are presented as n (%), mean ± standard deviation.

* *n* = 175 (2 missing)

**n = 172 (5 missing)

***n = 156 (only patients who reached day 30 were included)

Abbreviations: *VRE* vancomycin-resistant *Enterococcus faecium*, *VSE* vancomycin-susceptible *E. faecium*, *ICU* intensive care unit

Even when potential confounders mirroring the severity of illness before BSI onset (day − 7 to day − 1) were considered by multivariate logistic regression analysis, we could not outline a difference of the mortality risk between the VRE and VSE groups. In contrast, the mean SOFA_Non-GCS_ score (from day − 7 to day − 1) was an independent and strong predictor for mortality either on day 30 or 90 post-bacteraemia onset. The relative increase of the mortality risk was 32.1% (95% confidence interval (CI) 14.1–53.1%) (day 30 post-BSI onset) and 17.9% (day 90 post-BSI onset), respectively, for each additional point in the SOFA_Non-GCS_ score. A similar finding was obtained for the combined endpoint, with an elevated risk for an unfavourable outcome at day 30 and 90 of 24.8 and 17.9%, respectively. As already shown in the univariate analysis, even after adjustment for confounders, vancomycin resistance was not identified as an independent risk factor for mortality or unfavourable outcome after 30 (Tables 3, 4) or 90 days (Additional file 1: Table S4 and Additional file 1:Table S5).

**Table 3:** Risk factors for death within 30 days after *Enterococcus faecium* bacteraemia (Cox regression; *n* = 177)

			Univariate			Multivariate		
Variables	Dead *n* = 21	Alive *n* = 156	HR	95% CI	*p*	HR	95% CI	*p*
Definitive infectious focus elimination								
Yes	6 (28.6)	40 (25.6)	0.85	0.33–2.20	0.745	0.50	0.19–1.37	0.181
No	15 (71.4)	116 (74.4)						
Start of adequate AB Therapy within < 48 h post-BSI onset								
Yes	14 (66.7)	90 (57.7)	1.47	0.59–3.64	0.407	1.03	0.39–2.74	0.952
No	7 (33.3)	66 (42.3)						
Mean SOFA_Non-GCS_ (day-7 until day-1 before BSI onset)	12.8 ± 3.8	6.8 ± 4.7	1.30	1.16–1.46	< 0.001	1.32	1.14–1.53	< 0.001
ICU stay between day-7 and BSI onset [days]	7.6 ± 1.2	5.0 ± 3.5	1.47	1.08–2.00	0.014	1.01	0.71–1-44	0.966
Bacteraemia								
VRE	6 (15.4)	33 (84.6)	1.49	0.58–3.84	0.409	1.80	0.64–5.03	0.264
VSE	15 (10.9)	123 (89.1)						

Data are presented as n (%) or mean ± standard deviation.

Abbreviations: *HR* hazards ratio, *CI* confidence interval, *AB* antibiotic therapy, *BSI* bloodstream infection, *SOFA* sequential organ failure assessment, *GCS* Glasgow coma scale, *VRE* vancomycin-resistant *Enterococcus faecium*, *VSE* vancomycin-susceptible *Enterococcus faecium*

**Table 4:** Factors associated with unfavourable outcomes 30 days after *Enterococcus faecium* bacteraemia (logistic regression)

			Univariate			Multivariate		
Variables	Patients with favourable outcome *n* = 95	Patients with unfavourable outcome *n* = 80	OR	95% CI	*p*	OR	95% CI	*p*
Definitive infectious focus elimination								
Yes	28 (29.5)	17 (21.2)	1.55	0.77–3.10	0.217	1.92	0.79–4.66	0.150
No	67 (70.5)	63 (78.8)						
Start of adequate AB Therapy within < 48 h post-BSI onset								
Yes	48 (50.5)	54 (67.5)	2.03	1.10–3.77	0.0241	1.62	0.73–3.60	0.238
No	47 (49.5)	26 (32.5)						
Mean SOFA_Non-GCS_ (day-7 until day-1 before BSI onset)	4.8 ± 3.8	10.8 ± 4.2	1.39	1.26–1.53	< 0.001	1.2%	1.10–1.41	< 0.001
ICU stay between day-7 and BSI onset [days]	3.7 ± 3.5	7.4 ± 1.8	1.55	1.34–1.78	< 0.001	1.24	1.03–1.49	0.023
Bacteraemia								
VRE	19 (48.7)	20 (51.3)	1.33	0.65–2.72	0.429	1.13	0.46–2.76	0.795
VSE	76 (55.9)	60 (44.1)						

Favourable outcome was defined as stay at home, on the ward or in a rehabilitation centre; unfavourable outcome was defined as death or ongoing need for ICU treatment.

Data are presented as n (%) or mean ± standard deviation.

*n = 175 (2 missing)

Abbreviations: *OR* odds ratio, *CI* confidence interval, *AB* antibiotic therapy, *BSI* bloodstream infection, *SOFA* sequential organ failure assessment, *GCS* Glasgow coma scale, *VRE* vancomycin-resistant *Enterococcus faecium*, *VSE* vancomycin-susceptible *Enterococcus faecium*

## Discussion

In this multivariate retrospective analysis on the outcome of patients with VRE- or VSE-BSI after OLT, vancomycin resistance was not identified to be a risk factor for mortality and was not associated with an increased 30- or 90-day mortality. This lack of an effect was confirmed even after adjustment for various potential confounders. Likewise, there was no association with the combined endpoint of an unfavourable outcome, which refers to death or prolonged need for ICU treatment.

To focus on this group of patients is relevant because of their notoriously high risk for VRE and it allows outcome analysis in a more homogenous cohort than in several other investigations, which included patients with broadly varying underlying diseases [24–27]. Mortality rates 30 days after VRE-BSI and VSE-BSI ranging from 11.0 to 46.4% and 9 to 38%, respectively, have been reported [24, 28–31]. Consistent with these data, mortality rates of 15.4 and 10.9% after VRE- and VSE-BSI were observed within the presented work. Similarly, 38.5% of VRE and 31.2% of VSE patients died after 90 days (*p* = 0.391).

To the best of our knowledge, three meta-analyses in this field evaluated the effect of VRE bacteraemia within the last two decades and attributed an increased risk of death to vancomycin resistance [12, 13, 32]. Two of these three meta-analyses [12, 32], and other studies that specifically focussed on liver transplant patients [10, 33], included data obtained in an era before effective antibiotic treatment for VRE bacteraemia (e.g., linezolid) was broadly available. Hence, these studies are not comparable to our results. Another problem of the above-mentioned meta-analyses is the inclusion of bacteraemia cases caused by different species of enterococci. This issue implicates a relevant risk of bias due to varying pathogenicity [34, 35] and virulence of *E. faecium* and *E. faecalis* and even other enterococcal species. A German study [36] reported increased in-hospital mortality in patients with *E. faecium* compared to *E. faecalis* BSI even after adjusting for underlying disease and vancomycin resistance. Similarly, even after adjustment for effective antibiotic treatment, Hayakawa et al. [37] observed impaired outcome following BSI due to vancomycin-resistant *E. faecium* compared to vancomycin-resistant *E. faecalis* strains. Prematunge et al. [13] already referred to this fact in their meta-analysis, stating that many observations on VRE outcomes might be based on differences in the causative species rather than resistance itself. Analogous to our study, Yoo et al. [38] analysed enterococcal BSI cases strictly focussing on *E. faecium*; however, their number of patients was very small and information about the time to adequate antibiotic therapy was lacking.

The meta-analysis of Prematunge et al. [13], who exclusively focussed on findings after the widespread use of linezolid, also derived a higher mortality risk due to vancomycin resistance from cohort studies (hazards ratio 1.80; 95% confidence interval 1.38–2.35). However, seven of the 12 included studies did not adjust for confounders; which is an important methodological flaw [14, 39]. Therefore, as the authors concede, these studies could not truly figure out whether differing mortality rates mirror the effect of resistance itself or rather different underlying conditions of patients affected by VRE versus VSE bacteraemia. Corroborating this assumption, studies that adjusted for potential confounders could not identify an effect of vancomycin resistance on mortality after enterococcal bacteraemia [25, 26, 29, 40, 41]. Our study was specifically designed to elaborate the mere impact of VRE on outcomes by meticulously addressing the influence of the underlying disease severity, including previous need of ICU treatment, adequacy and timeliness of antibiotic treatment and control of the infectious focus.

Most studies, that examined the underlying severity of illness in this field, relied on scores on the day of BSI onset. This approach is problematic because values assessed on the first day of bacteraemia might already reflect the effects of the BSI rather than the patients’ acute state of illness independent from and prior to bacteraemia. To circumvent this problem in our study, SOFA_Non-GCS_ Scores from day − 7 until the day before BSI onset were collected. Multivariate analysis confirmed the overwhelming impact of the acute state of illness on the patients‘outcome. Similarly, Cheah et al. [25], identified a strong association between the underlying status of the patient (as assessed by charlson comorbidity index) before enterococcal BSI and death. This appraisal is corroborated by the finding that interventions to increase arterial graft perfusion were performed more frequently in VRE patients, reflecting that a higher proportion of them were exposed to a critically compromised graft perfusion predisposing to chronic ischemic bile duct damage and a high risk of chronic hepatic infections, including liver abscess and cholangitis [42]. This was again mirrored by the distribution of infectious focuses with a broad majority of intra-abdominal sources of enterococcal bacteraemia and the corresponding increased mortality rates in this patient subgroup.

Time to the application of an effective antimicrobial substance is crucial for successful BSI treatment. Recent analyses of enterococcal blood stream infections showed worse outcome when adequate antibiotic treatment was delayed [25, 43, 44]; Zasowski et al. [44] and Vergis et al. [11] identified a breakpoint of 48 h after BSI onset. Several studies that supposed a worse outcome associated with VRE compared to VSE bacteraemia [27, 28, 30] adjusted for neither adequacy nor timeliness of antibiotic therapy. Therefore, this aspect was specifically addressed in the multivariate analysis in the present work. Definitive infectious focus elimination could only be achieved in a minority of patients in both groups mirroring the complex underlying anatomical and vascular disturbances in this selected group of patients, which is a typical scenario predisposing for enterococcal bacteraemia [45]. This aspect is another confounder that is rarely addressed in enterococcal bacteraemia studies. The finding, that the 30- and 90-day mortality rates of VRE (15.4 and 38.5%) and VSE patients (10.9 and 31.2%) were nevertheless moderate might be explained by the fact, that measures to control the infectious source were systematically performed in all patients, even when complete eradication was reached only in the minority of them. Moreover, the moderate mortality rates might also mirror the relatively low virulence of enterococci compared to gram-negative rods or *Staphylococcus aureus* [46].

After carefully adjusting for confounding factors, our results suggest that not vancomycin resistance itself, but the severity of illness before *E. faecium* BSI onset, was associated with death and an unfavourable outcome. Given these results, one could put contact isolation policies in question. The body of evidence is steadily increasing, that –provided the compliance with standard hygiene and routine cleaning procedures is high- isolation measures due to VRE can be suspended without harm and even reduce other non-infectious adverse events [47, 48]. International recommendations for the prevention of colonisation and infection of VRE show a wide heterogenicity and no consensus, probably due to a differing prevalence and varying structures of international health care systems [49, 50].

The proportion of linezolid resistant *Enterococcus faecium* isolates was nearly the same in the VRE- and the VSE-group (2.6 and 2.2%, respectively). Nevertheless, vancomycin-resistance is a main-driver of linezolid use contributing to selection pressure in favour of linezolid-resistant isolates. Therefore, another important part of preventive strategies are antibiotic stewardship programs.

This study has several limitations. First, it was a retrospective single-centre study. Therefore, the generalisability of our findings to other settings or patient cohorts must be considered cautiously. Second, because breakpoints for daptomycin susceptibility testing of enterococci are not provided by the European Committee on Antimicrobial Susceptibility Testing (EUCAST), the clinical isolates in this study were not routinely tested. Therefore, we have no data on potential therapeutic alternatives beyond linezolid to treat patients with VRE bacteraemia. Recent studies and meta-analyses that compared daptomycin and linezolid could not provide a definitive answer to this question, but some studies tend to favour daptomycin [51–53]. Third, we did not provide information about the genotypes of the included *E. faecium* strains. Researchers who focus on the influence of different genotypes state that the VanB genotype is associated with increased length of stay compared to the VanA genotype [54]. This topic requires further investigation. Fourth, immunosuppression is a potentially important factor in this population. Since we do not have individual data about the immunosuppressive medication in every patient, we were not able to consider this aspect in the multivariate analysis. This could have influenced our results and is another limitation of this study.

## Conclusion

In summary, our multivariate analysis identified the mean-SOFA_Non-GCS_ scores before BSI onset as the only strong and independent predictor of mortality. Each additional point increased the likelihood of death after 30 days by 32.1% (95% CI 14.1–53.1%) and also the potential for an unfavourable outcome by day 30 and 90 post-BSI. In contrast, in an era of broadly available effective treatment, vancomycin resistance had no impact on mortality or a poor outcome in patients with *E. faecium* BSI after OLT.

## Supplementary information

**Additional file 1: Table S1.** Polymicrobial blood stream infections and identified Co-pathogens in study subjects. **Table S2**. Antibiotic therapy used to treat the study subjects **Table S3.** Interventions from blood stream infection (BSI) until day 30 **Table S4.** Risk factors for death within 90 days after *Enterococcus faecium* bacteraemia (Cox regression). **Table S5.** Factors associated with unfavourable outcomes 90 days after *Enterococcus faecium* bacteraemia (logistic regression). **Table S6.** Source of infection and related outcome in patients with vancomycin-resistant *Enterococcus faecium* (VRE) **Table S7.** Source of infection and related outcome in patients with vancomycin-susceptible *E. faecium* (VSE) bacteraemia.

## Data Availability

All data generated or analysed during this study are included in this published article [and its supplementary information files] Original data are available from the corresponding author upon reasonable request.

## Abbreviations

BDA: Biliodigestive anastomosis
BSI: Blood stream infections
CI: Confidence interval
ERCP: Endoscopic retrograde cholangiopancreatography
EUCAST: European Committee on Antimicrobial Susceptibility Testing
HR: Hazard ratio
ICU: Intensive care unit
MDRO: Multidrug-resistant organisms
MELD: Model of end stage liver disease
OLT: Orthotopic liver transplantation
PCR: Polymerase chain reaction
PTCD: Percutaneous transhepatic cholangiodrainage
SOFA: Sequential organ failure assessment score
VRE: Vancomycin-resistant enterococci
VSE: Vancomycin-susceptible enterococci
