# Supplementary material for: Does vancomycin resistance increase mortality in Enterococcus faecium bacteraemia after orthotopic liver transplantation? A retrospective study
Source: Antimicrob Resist Infect Control. 2020 Jan 31;9:22. doi: 10.1186/s13756-020-0683-3 (PMC6995054; doi:10.1186/s13756-020-0683-3)
Supplement: Supplementary file 1 — Additional file 1: Table S1. Polymicrobial blood stream infections and identified Co-pathogens in study subjects. Table S2. Antibiotic therapy used to treat the study subjects Table S3. Interventions from blood stream infection (BSI) until day 30 Table S4. Risk factors for death within 90 days after Enterococcus faecium bacteraemia (Cox regression). Table S5. Factors associated with unfavourable outcomes 90 days after Enterococcus faecium bacteraemia (logistic regression). Table S6. Source of infection and related outcome in patients with vancomycin-resistant Enterococcus faecium (VRE) Table S7. Source of infection and related outcome in patients with vancomycin-susceptible E. faecium (VSE) bacteraemia. [file 13756_2020_683_MOESM1_ESM.docx]

**Supplementary Tables**

**Table S1:** Polymicrobial blood stream infections and identified Co-pathogens in study subjects.

|  | **VRE patients**  **n = 39** | **VSE patients**  **n = 138** |
| --- | --- | --- |
| **Polymicrobial blood stream infections*** | **5 (12.8)** | **12 (8.7)** |
| **Isolated Co-pathogens together with *E. faecium*** | *Enterococcus faecalis* | *Candida krusei* |
|  | *Stenotrophomonas maltophilia* | *Escherichia coli* |
|  | *Raoultella planticola* | *Enterococcus. faecalis* |
|  | *Klebsiella pneumoniae* | *Citrobacter braakii* |
|  | *Enterococcus faecalis* +  *Enterococcus casseliflavus* | *Pseudomonas aeruginosa* +  *Enterobacter cloacae* |
|  |  | *Serratia marcescens* |
|  |  | *Pseudomonas aeruginosa* |
|  |  | *Escherichia coli* |
|  |  | *Neisseria* species |
|  |  | *Enterococcus faecalis* |
|  |  | *Escherichia coli* |
|  |  | *Klebsiella pneumoniae* |

*p=0.440

**Table S2**: Antibiotic therapy used to treat the study subjects.

|  | **VRE**  **n = 39** | **VSE**  **n = 138** | ***p*** |
| --- | --- | --- | --- |
| Targeted Antibiotic therapy |  |  |  |
| Imipenem | 0 (0) | 2 (1.5) |  |
| Vancomycin | 0 (0) | 88 (63.8) |  |
| Teicoplanin | 0 (0) | 1 (0.7) |  |
| Tigecycline | 6 (15.4) | 4 (2.9) |  |
| Linezolid | 31 (79.5) | 31 (22.5) |  |
| Other (Imipenem+Vancomycin) | 0 (0) | 1 (0.7) |  |
| No therapy | 2 (5.1) | 11 (8.0) | 0.548 |
| Empirical therapy | 6 (16.2) | 22 (17.3) | 0.875 |
| Start of adequate therapy <48h post-BSI onset [yes] | 21 (53.9) | 83 (60.1) | 0.481 |
| Adequate therapy for at least 5 days [yes] | 33 (84.6) | 125 (90.6) | 0.288 |

Data are presented as n (%) or mean ± standard deviation.

Abbreviations: VRE, vancomycin-resistant enterococci; VSE, vancomycin-sensitive enterococci; BSI, bloodstream infection.

**Table S3:** Interventions from blood stream infection (BSI) until day 30

|  | **All Patients**  **(n=177)** | **VRE**  **n = 33** | **VSE**  **n = 123** | ***p*** |
| --- | --- | --- | --- | --- |
| Interventions (BSI to day 30) to: |  |  |  |  |
| increase arterial perfusion to the liver | 156* | 0.3 ±0.6 | 0.1 ±0.4 | 0.124 |
| drain an intraabdominal abscess | 156 | 0.6 ±0.7 | 0.5 ±0.8 | 0.094 |
| improve bile flow | 156 | 1.0 ±1.3 | 0.8 ±1.0 | 0.916 |
| Laparotomies | 156 | 0.4 ±0.7 | 0.5 ±1.1 | 0.646 |
| Re-OLT | 156 | 0.1 ±0.2 | 0.1 ±0.2 | 0.940 |
| Total interventions | 156 | 2.3 ±1.8 | 1.9 ±1.8 | 0.197 |

Data are presented as n (%) or mean ± standard deviation.

*n= 156 (only patients who were alive by day 30 were included)

Abbreviations: VRE, vancomycin-resistant enterococci; VSE, vancomycin-sensitive enterococci; OLT, orthotopic liver transplantation.

**Table S4:** Risk factors for **death within 90 days after** *Enterococcus faecium* bacteraemia (Cox regression).

|  | | | **Univariate** | | | **Multivariate** | | |
| --- | --- | --- | --- | --- | --- | --- | --- | --- |
| **Variables** | **Dead**  **n = 58** | **Alive**  **n = 119** | **HR** | **95% CI** | ***p*** | **HR** | **95% CI** | ***p*** |
| Definitive infectious focus elimination |  |  |  |  |  |  |  |  |
| Yes | 11 (19.0) | 35 (29.4) | 1.59 | 0.83 – 3.07 | 0.165 | 1.47 | 0.73 – 2.96 | 0.280 |
| No | 47 (81.0) | 84 (70.6) |  |  |  |  |  |  |
| Start of adequate AB Therapy within < 48h post-BSI onset |  |  |  |  |  |  |  |  |
| Yes | 39 (67.2) | 65 (54.6) | 1.56 | 0.92 – 2.76 | 0.095 | 1.17 | 0.65– 2.09 | 0.605 |
| No | 19 (32.8) | 54 (45.4) |  |  |  |  |  |  |
| Mean SOFA_Non-GCS_ (day-7 until day-1 before BSI onset) | 10.8 ±4.5 | 5.9 ±4.4 | 1.21 | 1.14 – 1.29 | <0.001 | 1.18 | 1.08 – 1.28 | <0.001 |
| ICU stay between day-7 and BSI onset [days] | 7.2 ±2.1 | 4.5 ±3.6 | 1.31 | 1.16 – 1.48 | <0.001 | 1.07 | 0.92 – 1.26 | 0.386 |
| Bacteraemia |  |  |  |  |  |  |  |  |
| VRE | 15 (38.5) | 24 (61.5) | 1.38 | 0.77 – 2.49 | 0.281 | 1.53 | 0.83 – 2.83 | 0.176 |
| VSE | 43 (31.2) | 95 (68.8) |  |  |  |  |  |  |

Data are presented as n (%) or mean ± standard deviation.

Abbreviations: HR, hazards ratio; CI, confidence interval; AB: antibiotic therapy; BSI: bloodstream infection; SOFA: sequential organ failure assessment; GCS: Glasgow coma scale; VRE: vancomycin-resistant Enterococcus faecium; VSE: vancomycin-susceptible E. faecium.

**Table S5:** Factors associated **with unfavourable outcomes 90 days** after *Enterococcus faecium* bacteraemia (logistic regression).

|  | | | **Univariate** | | | **Multivariate** | | |
| --- | --- | --- | --- | --- | --- | --- | --- | --- |
| **Variables** | **Patients with favourable outcome**  **n = 91** | **Patients with unfavourable outcome**  **n = 81** | **OR** | **95% CI** | ***p*** | **OR** | **95% CI** | ***p*** |
| Definitive infectious focus elimination |  |  |  |  |  |  |  |  |
| Yes | 26 (28.6) | 17 (21.0) | 1.51 | 0.75 – 3.04 | 0.253 | 1.87 | 0.78 – 4.49 | 0.162 |
| No | 65 (71.4) | 64 (79.0) |  |  |  |  |  |  |
| Start of adequate AB Therapy within < 48h post-BSI onset |  |  |  |  |  |  |  |  |
| Yes | 46 (50.6) | 55 (67.9) | 2.07 | 1.11 – 3.85 | 0.022 | 1.78 | 0.83 – 3.80 | 0.137 |
| No | 45 (49.5) | 26 (32.1) |  |  |  |  |  |  |
| Mean SOFA_Non-GCS_ (day-7 until day-1 before BSI onset) | 5.0 ±3.9 | 10.5 ±4.6 | 1.32 | 1.21 – 1.44 | <0.001 | 1.23 | 1.09 – 1.39 | 0.001 |
| ICU stay between day-7 and BSI onset [days] | 3.8 ±3.6 | 7.0 ±2.4 | 1.38 | 1.23 – 1.54 | <0.001 | 1.14 | 0.96 – 1.35 | 0.134 |
| Bacteraemia |  |  |  |  |  |  |  |  |
| VRE | 20 (51.3) | 19 (48.7) | 1.09 | 0.53 – 2.22 | 0.817 | 0.90 | 0.38 – 2.12 | 0.812 |
| VSE | 71 (53.4) | 62 (46.6) |  |  |  |  |  |  |

Favourable outcome was defined as stay at home, on the ward or in a rehabilitation centre; unfavourable outcome was defined as death or ongoing need for ICU treatment

Data are presented as n (%) or mean ± standard deviation.

*n=172 (5 missing)

Abbreviations: OR, odds ratio; CI, confidence interval; AB: antibiotic therapy, BSI: bloodstream infection, SOFA: sequential organ failure assessment, GCS: Glasgow coma scale, VRE: vancomycin-resistant Enterococcus faecium, VSE: vancomycin-susceptible E. faecium.

**Table S6:** Source of infection and related outcome in patients with vancomycin-resistant *Enterococcus faecium* (VRE)

| **Source of infection** | **VRE patients**  **(n=39)** | **Crude 30d mortality** |
| --- | --- | --- |
| Abdominal | 36/39 (92.3%) | 5/36 (13.9%) |
| Catheter related | 0/39 (0%) | - |
| Urinary tract infection | 1/39 (2.6%) | 0/1 (0%) |
| Others | 0/39 (0%) | - |
| Unknown origin | 2/39 (5.1%) | 1/2 (50%) |

Abbreviations: VRE, vancomycin-resistant enterococci;

Data are presented as n (%) or mean ± standard deviation.

**Table S7:** Source of infection and related outcome in patients with vancomycin-susceptible *E. faecium* (VSE) bacteraemia.

| **Source of infection** | **VSE patients**  **(n=138)** | **Crude 30d mortality** |
| --- | --- | --- |
| Abdominal | 114/138 (82.6%) | 13/114 (11.4%) |
| Catheter related | 1/138 (0.7%) | 1/1 (100%) |
| Urinary tract infection | 2/138 (1.4%) | 0/2 (0%) |
| Others | 1/138 (0.7%) | 0/1 (0%) |
| Unknown origin | 20/138 (14.5%) | 1/20 (5%) |

Abbreviations: VSE, vancomycin-sensitive enterococci;

Data are presented as n (%) or mean ± standard deviation.
